# Greener healing: sustainable nanotechnology for advanced wound care

**DOI:** 10.1186/s11671-024-04061-1

**Published:** 2024-08-13

**Authors:** Deepinder Sharda, Komal Attri, Diptiman Choudhury

**Affiliations:** 1 School of Chemistry and Biochemistry, Thapar Institute of Engineering and Technology, Patiala, Punjab 147004 India; 2 Thapar Institute of Engineering and Technology-Virginia Tech (USA) Centre of Excellence in Emerging Materials, Thapar Institute of Engineering and Technology, Patiala, Punjab 147004 India

**Keywords:** Wound healing, Polyphenols, Cytokines, Anti-inflammatory agents, Nanoparticles

## Abstract

Wound healing involves a carefully regulated sequence of events, encompassing pro-inflammatory and anti-inflammatory stages, tissue regeneration, and remodeling. However, in individuals with diabetes, this process gets disrupted due to dysregulation caused by elevated glucose levels and pro-inflammatory cytokines in the bloodstream. Consequently, the pro-inflammatory stage is prolonged, while the anti-inflammatory phase is delayed, leading to impaired tissue regeneration and remodeling with extended healing time. Furthermore, the increased glucose levels in open wounds create an environment conducive to microbial growth and tissue sepsis, which can escalate to the point of limb amputation. Managing diabetic wounds requires meticulous care and monitoring due to the lack of widely available preventative and therapeutic measures. Existing clinical interventions have limitations, such as slow recovery rates, high costs, and inefficient drug delivery methods. Therefore, exploring alternative avenues to develop effective wound-healing treatments is essential. Nature offers a vast array of resources in the form of secondary metabolites, notably polyphenols, known for their antimicrobial, anti-inflammatory, antioxidant, glucose-regulating, and cell growth-promoting properties. Additionally, nanoparticles synthesized through environmentally friendly methods hold promise for wound healing applications in diabetic and non-diabetic conditions. This review provides a comprehensive discussion and summary of the potential wound-healing abilities of specific natural polyphenols and their nanoparticles. It explores the mechanisms of action underlying their efficacy and presents effective formulations for promoting wound-healing activity.

**Graphical abstract:**

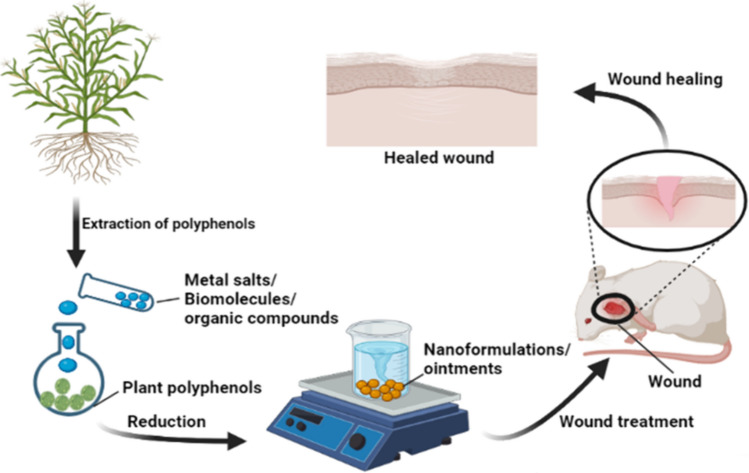

## Introduction

Despite the extensive development and management in treating injuries, chronic wounds always remained a challenge before scientists and clinicians. The wound is the loss of tissue continuity either chemically, electrically, mechanically, or due to radiation exposure [1, 2] and can be internal or external and acute or chronic [3, 4]. Chronic wounds pose a severe health burden and are a significant clinical problem when combined with diabetes or other vascular ailments, particularly in older people [5]. Worldwide, around 40 million people have chronic wounds, which may last for three months as compared to acute injuries, which last for 14 days, and their treatment puts an economic burden globally as the international market is expected to rise to 24.8 billion in 2024 from 19.8 billion in 2021 [6–8].

The wound healing process helps restore skin integrity by natural or artificial means and has three major phases: hemostasis/ inflammation, proliferation, and remodeling. At each stage of wound healing, different types of cells, such as fibroblasts, leukocytes, and keratinocytes, under the control of chemokines, cytokines, and various growth factors interact with each other and promote healing [9, 10]. During hemostasis, the platelets activate and release chemokines and other growth factors, forming a clot. After coagulation, the platelets help to create a barrier against microorganisms, and the matrix organization is responsible for migrating cells [11]. The cells and connective tissue, alongside other factors, such as cytokines, growth factors, and angiogenesis factors, accumulate in the proliferation phase [12]. During the remodeling phase, the extracellular matrix is resynthesized to maintain a balance between the death of existing cells and the formation of new cells [13]. The wound healing process is a cascade of interlinked events that typically induce the release of various pro-inflammatory cytokines such as tumor necrosis factors (TNFs), interferon-gamma (IFN-γ), interleukin-1 (IL-1), IL-6, IL-12, and IL-18 [14, 15]. The transition of pro-inflammatory cytokines to anti-inflammatory cytokines, such as transforming growth factor-beta (TGF-β), Interferon-alpha (IFN-α), IL-4, IL-10, IL-11, and IL-13, is necessary for wound healing as they stimulate the formation of extracellular matrix, fibrinogen synthesis, and cell division [16, 17].

Chronic or diabetic wounds are characterized by the persistent expression of the pro-inflammatory cytokines, which cause an interruption in normal wound healing and result in non-healing or impaired healing of wounds [18, 19]. Diabetes further leads to several complications causing clinical morbidity, which includes infection, gangrene, amputations, ulcers, and wound dehiscence after the operation, etc. [20–22]. Further, under hyperglycemic conditions, there is an alteration in cellular Na + /K + ATPase activity and an increase in protein kinase activity, which alters hormone receptor turnover and cell growth [23]. Additionally, advanced glycosylation end products are formed during hyperglycemic conditions, increasing oxidative stress, affecting collagen degradation, making cell walls more permeable, and causing dysfunctioning of endothelial cells and extracellular matrix [23]. Several abnormalities in different wound healing stages in humans and animals include delayed cellular infiltration and granulation tissue formation, reduced angiogenesis, and decreased and disorganized collagen formation [24, 25]. Therefore, it is quite challenging to take proper care of diabetic wounds, and there is a need to develop new therapies that improve healing efficiency in diabetic patients. So, emphasis must be placed on using compounds that not only cure the wound but also do not cause any other harm to the body [26, 27]. A wide range of drugs, gels, and formulations are available for treating both normal and diabetic wounds by exhibiting anti-inflammatory and antimicrobial effects [28–31]. These drugs possess positive and negative aspects that directly or indirectly affect the patient’s quality of life. People nowadays prefer products developed using natural products with little or no side effects, low toxicology profiles, and cost-effectiveness.

Various natural formulations have been used for many decades in distinct parts of the world, including India, China, and Africa, in one way or another through different medicinal systems, including Ayurveda, homeopathy, Unani, etc. These formulations primarily comprise plant secondary metabolites, including phenolics, saponins, flavonoids, terpenes, terpenoids, sterols, and sphingolipids [32]. Being driven by natural products, these formulations are gaining huge interest due to their lack of side effects when compared with synthetic medicines. Surgical wounds take more time to heal, and patients take different medications, of which the antibiotics help to get rid of infections, but other medications, such as steroids, decrease the epithelialization and neovascularization rate, prevent wound contraction, and decrease the tensile strength of the wound. Non-steroidal anti-inflammatory drugs have been found to suppress the inflammatory response by causing vasoconstriction [33]. Therefore, there is a need to develop novel formulations using green methods and products. One such compound group is polyphenols, a broad family of natural products. They significantly impact skin ailments by exhibiting anti-oxidant, anti-inflammatory, and anti-microbial activity apart from anti-aging and skin whitening effects [34]. Their antimicrobial potential is not fully explored yet but is believed to be in association with the disintegration of the bacterial cell wall via hydrophobic components of phenolic compounds, the changes in intracellular functions by hydrogen binding of these bioactive compounds to enzymes, or by the modification of the cell wall rigidly with integrity losses due to different interactions with the cell membrane [35]. The lipophilic character of polyphenols enhances antimicrobial activity. Based on the phenol unit present in each polyphenol, they exhibit a wide range of applications in tissue engineering, including reducibility for scavenging free radicals, higher affinity towards different proteins through specific or unspecific interactions, interaction ability with various receptors, signal transduction modulation, regulation of enzyme activity, devitalization of micro-organisms and cross-linking of biomolecules thus, making them one of the promising and leading herbal material for wound healing [36–39]. Despite numerous positive aspects, polyphenols and other plant products are not being widely employed for wound healing applications due to various limitations associated with their efficient transport across the human body, due to their low stability and poor water solubility, low absorption, reduced bioavailability, and insufficient delivery. Further, polyphenolic compounds have short half-lives, lower biocompatibility, and are susceptible to environmental conditions such as moisture, heat, oxygen, and light irradiation [40]. Here, the researchers make use of nanotechnology to overcome these disadvantages.

Nanotechnology involves the synthesis of nanomaterials at 1–100 nm scale by manipulating the structure at the atomic level and utilizing their critical properties at the nanoscale. These nanomaterials possess a large surface area to volume ratio, which increases their catalytic and biological activity, thermal conductivity, and non-linear optical performance [41]. Nanomaterials have unique optical, electrical, and magnetic properties, improving their target specificity and drug delivery and giving them an upper hand over traditional therapeutics. All these factors significantly contribute to the enormous interest of researchers globally in developing and exploring futuristic wound healing materials using polyphenols [42].

This review article primarily focusses on the potential of polyphenols in developing nanomaterials for their role in normal and diabetic wound healing and highlights the positive aspects of polyphenol-based nanomaterials over traditional intake of polyphenols in the form of food supplements or directly as herbal medications. Further, we will focus on the tremendous potential of developing herbal nano-formulations for better drug delivery, biocompatibility, target specificity, and cost-effectiveness.

### Polyphenols and their classification

Chemically, polyphenols are molecules having one or more aromatic rings with two or more hydroxyl groups and can occur either in free forms or in conjugation with other compounds such as acids, sugars, and other biomolecules, which can either be soluble or insoluble in water [43]. These compounds provide vegetables and fruits with colors and sensory characteristics that help pollinate and disperse seeds. Further, they protect plants from UV radiations, oxidative stress, pathogens, and predators and aid their growth and reproduction [44, 45]. Polyphenols are mainly derived from plants, especially from fruits and vegetables, and act as secondary metabolites. Further, these metabolites play a crucial role in fighting against certain diseases ranging from diabetes, wound care, Parkinson’s disease, Alzheimer’s disease, and so on [46, 47]. Their biological properties depend on various factors such as concentration in food, polymerization degree, interaction with other molecules, and bio-accessibility after ingestion [48]. More than 8000 polyphenols have been identified to date and are divided into four classes: phenolic acids, flavonoids, stilbenes, and tannins, depending upon the number of phenol rings present and the binding properties of these ring structures.

#### Phenolic acids

Phenolic compounds have at least one hydrogen substituted by hydroxyl groups in the aromatic ring [49] and are further divided into hydroxybenzoic acids (HBAs) and hydroxycinnamic acids (HCAs), respectively derived from nonphenolic molecules of benzoic and cinnamic acid [50]. The HBAs comprise C6–C1 in their general structure with some changes in the basic structure, including methoxylation and hydroxylation of their aromatic rings [51]. HBAs can be salicylic acid, 4-hydroxybenzoic acid, protocatechuic acid, gentisic acid, vanillic acid, syringic acid, gallic acid, ellagic acid, and hexahydroxydiphenic acid [44]. On the other side, HCAs have C6–C3 in their basic structure with a double bond in the side chain that may have a cis or a trans configuration. They are usually found in monomeric, dimeric, and polymeric forms in food or can be present as condensates with different groups such as alcohols, hydroxy acids, amines, or mono/disaccharides producing esters [52]. They have the ability to scavenge other radicals, including hydroxyl radicals, superoxide anion radicals, various organic radicals, and peroxy radicals, and thus, exert antioxidant activity. They can change the cell signaling pathway and play a role as a chain-breaking antioxidant and reducing agent [53]. Ferulic acid and caffeic acid protect human skin against erythema induced by UV irradiation [54]. Sinapic acid exhibits anti-inflammatory potential; p-coumaric acid helps inhibit low-density lipoprotein (LDL) oxidation and decrease LDL cholesterol levels [53].

#### Flavonoids

Flavonoids comprise the largest group of polyphenolic compounds, with more than 6000 compounds out of 8000 polyphenols found in plant food. They have a pretty low molecular weight and consist of a 15-carbon skeleton in the form of C6–C3–C6, having distinct substitutions, unsaturation degree, and arrangement of the basic skeleton, thus resulting in various categories. They consist of two rings, A and B, which are aromatic and are joined by another heterocyclic ring, C [55]. They are subdivided into flavones, chalcones, isoflavones, and flavonols [56]. Flavonoids are extracted from several parts of plants and are utilized by vegetables in growth and defense mechanisms [57]. Further, they assist in pollination by providing aroma and color to fruits and vegetables, help in seed dispersal, germination, growth, and development, and protect them from stress and UV irradiation [58]. They can modulate enzyme functions at the cellular level and have various anti-oxidant, anti-inflammatory, anti-cancerous, and anti-mutagenic properties, making them even more helpful [56].

#### Stilbenes

Stilbenes are mainly obtained from dead bark tissue and are rarely found in live bark tissue. The carbon skeleton is arranged as C6–C2–C6, having a diphenylethylene backbone [59]. These are also called phytoalexins due to their role in coping with stress conditions such as fungal attacks, UV radiations, and other mechanical injuries [59, 60]. They exist in two isomeric forms: E- stilbene, which is sterically unhindered, and Z-stilbene, which is sterically hindered, thus becoming more and less stable, respectively [61]. They are used in various applications, including dye manufacturing, optical brighteners manufacturing, and as a scintillator and luminescent material. They are also anti-oxidant, anti-cancerous, anti-inflammatory, antimicrobial, and vasoprotective plant polyphenols [61, 62].

#### Tannins

Tannins are commonly known as tannic acid. They have many hydroxyls or other functional groups and are thus obtained in their ester forms. Their molecular weight ranges from 500 to 3000 Da, are water-soluble and form insoluble complexes with proteins, gelatin, and alkaloids. Tannic acids play a role in interactions among plants and their ecosystems and can act as antimicrobial agents or against herbivores [63]. Based on their properties, they are further divided into hydrolyzable tannins consisting of three subtypes: gallotannines, ellagitannins, complex tannins, and nonhydrolyzable forms (condensed tannins) having a shortened carbon chain [64]. Tannins show toxicity against bacteria, fungi, and yeast, which have been known for many years [65]. Due to their bioactivity and successful clinical trials, they play an essential role in the pharmaceutical and nutraceutical industry [66].

### Polyphenols and their potential role in skin care and wound healing

Polyphenols exhibit excellent anti-oxidant properties and reduce oxidative stress and inflammatory responses, which play a crucial role in tissue repair in an ordered manner [67]. Based on phenol units present, polyphenols exhibit innate reducibility for scavenging free radicals and possess very high affinity towards different proteins through specific or unspecific interactions based on which they can interact with numerous receptors, help in the modulation of signal transduction, regulate enzymatic activity, assist in cross-linking biomacromolecules and devitalize micro-organisms [39].

### The anti-oxidant potential of polyphenols

Free radical generation, an integral part of cell metabolism, is markedly elevated during inflammatory response to wounds. Reactive oxygen species (ROS) are generated during cellular damage, necrosis, excessive neutrophil infiltration, and hypoxic conditions initiated by injuries [68]. Polyphenols possess huge anti-oxidant potential against the damage caused to the endogenous defense system of the skin by chronic wounds and assist in restoring redox imbalance by neutralizing harmful free radicals along with reactive oxygen species [6]. They suppress the peroxidation of lipids, decrease the level of NO and H_2_O_2_ production, and are found to interact directly with enzymes and receptors involved in the signal transduction process, ultimately modulating the redox status and improving cell survival [34]. Generally, polyphenols are found to follow three distinct mechanisms for scavenging ROS. The first one is the transfer of hydrogen atoms to the free radical *R.* by O–H bond homolysis, and the factors that are useful in weakening the O–H bond and have low bond dissociation energy show maximum antioxidant potential. The second one is the single electron transfer from the polyphenolic cation to free radical *R.* and is dependent upon the ionization potential of the polyphenolic moiety. Lower the ionization potential faster the electron transfer and free radical scavenging. The third one is polyphenols’ chelation of metal ions, making them inactive and preventing their involvement in radical-generated reactions [69, 70].

### Anti-microbial potential of polyphenols

Antibiotic resistance is becoming a global issue affecting multiple areas, so finding new alternatives to eliminate antibiotic-resistant bacterial strains is essential. Polyphenols are considered safe alternatives to chemical derivatives as antimicrobial agents, and various studies have been reported on their potential antimicrobial action against pathogenic bacterial strains [71]. Although the exact mechanism of their antimicrobial action is not yet known, but the possible mechanism includes the action of hydrophobic components of polyphenol in the disintegration of bacterial walls, hydrogen binding of polyphenols with enzymes leads to intracellular function alterations, and the loss of cell wall rigidity, loss of integrity due to interactions with the cell membrane [35]. Thus, they mainly act by rupturing the cell membrane, defecting the nucleic acids, depleting the adenosine triphosphate (ATP), or decaying proton motive forces [72]. The polyphenols with enhanced lipophilic characters show higher antimicrobial action [73].

### Anti-inflammatory potential of polyphenols

**Fig. 1:**
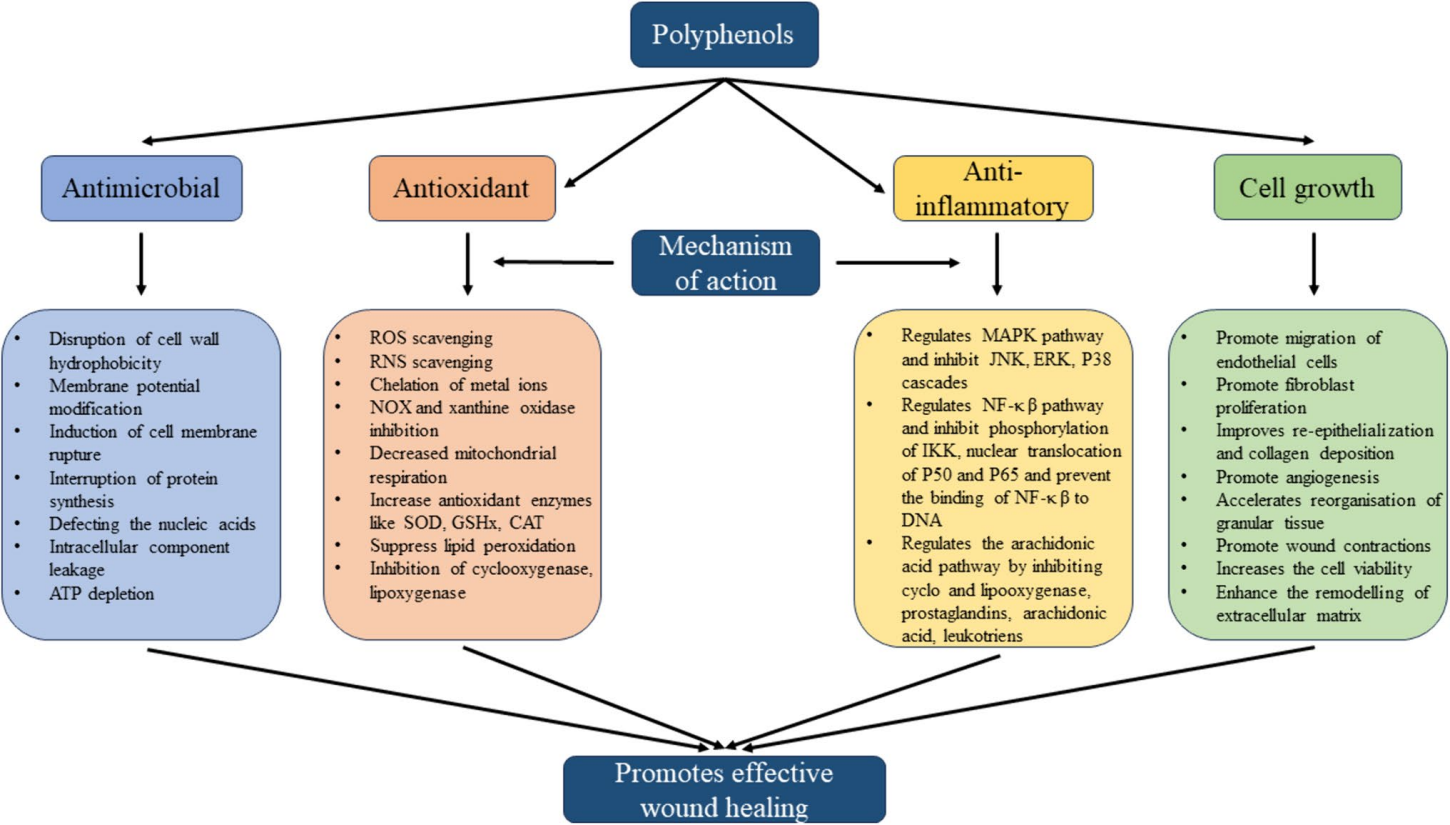
The figure describes the role of polyphenols as antioxidant, anti-inflammatory, anti-microbial, and cell growth-promoting agents, which are essential for effective wound healing by modulating multiple signaling pathways

Polyphenols are known to exhibit anti-inflammatory properties by targeting multiple inflammatory components. They suppress the proinflammatory gene expression and toll-like receptors and inhibit the enzymes required for producing eicosanoids. They can alter the expression of different pro-inflammatory genes, such as cytokines, lipoxygenase, and nitric oxide synthases cyclooxygenase, thus regulating inflammatory signaling [74]. Further, they suppress the macrophages that initiate inflammation by producing pro-inflammatory cytokines. They decrease the production of IL-6, TNF-α, and IL-1-β, and inhibit cyclooxygenase-2 (Cox-2), inducible nitric oxide synthase (iNOS) responsible for inflammation [75]. Polyphenols also directly or indirectly represses the lipoxygenase (LOX), IKK (inhibitor of kappa kinases), and MAPK (mitogen-activated protein kinase), which are crucial enzymes for inflammation. They downregulate specific pathways responsible for inflammatory action, including STAT-3, NF-κ β, and TLR-2 and 4 [76]. The potential role of polyphenols as antioxidant, antimicrobial, anti-inflammatory, and cell growth-promoting agents is crucial for effective wound healing by modulating multiple factors, as shown in Fig. 1.

### Polyphenol-derived nano-formulations and their synthesis

**Fig. 2:**
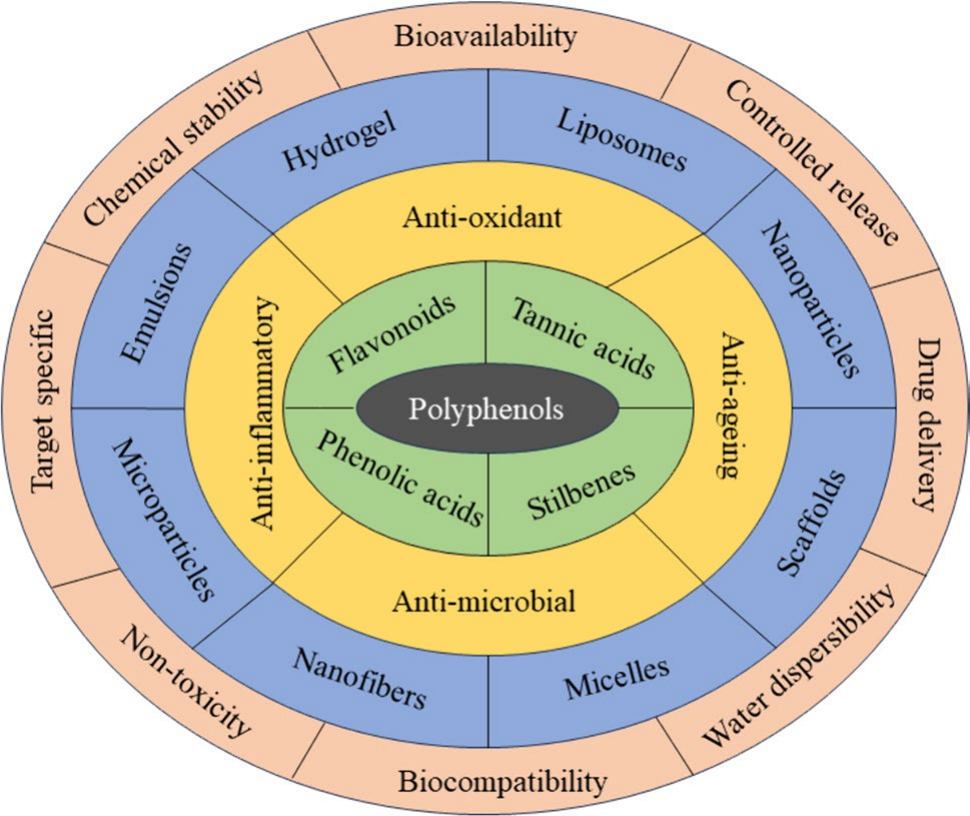
The given figure gives an overview of the classification of polyphenols, their role in skin healing, multiple nano-formulations obtained from polyphenols, and their advantages over other formulations used for wound healing

As discussed earlier, it becomes evident that polyphenols have considerable potential in treating various ailments, including wounds and injuries, depending upon their multiple beneficial factors, but certain factors hinder their in vitro*, *in vivo*,* and clinical applications. The major factors include their low solubility, low permeability across the cell walls, and, eventually, low bioavailability. Nano-formulations come into play to overcome these limitations as they have unique physicochemical properties, including high drug loading and protection efficiency, higher target penetration ability, and controlled drug release potential [77]. Various categories of nano-formulations are being synthesized these days, including metallic nanoparticles, solid lipid nanoparticles, liposomes, niosomes, protein-based formulations, nanospheres, nanoclusters, adhesive gel or cream ointments, scaffolds carrying cells and emulsions [78]. There are various means for synthesizing nanoparticles, including physical, chemical, and green methods [79, 80]. Those methods, which are environment-friendly and energy efficient, have a low cost of synthesis and give higher yield, are preferred [32]. Some of the most common polyphenolic nano-formulations and their advantages and synthesis methods are presented comparatively in Table 1 and shown in Fig. 2. The scalability and cost-effectiveness of all the synthesis methods discussed in Table 1 depend upon multiple factors. The cost of raw materials used affects the overall cost-effectiveness of synthesis, as most of the polyphenols are derived from nature. Thus, the cost is low, but sometimes the synthesis involves multiple-step reactions or expensive equipment, which increases the cost of production. Purification and characterization techniques ensure the end product’s efficacy and quality and add to the overall cost. However, if the synthesized formulation is stable and has an extended shelf-life, it will cut costs by decreasing the need for frequent production and storage. Additionally, the green methods that minimize waste generation, energy consumption, and production of harmful chemicals will reduce environmental compliance costs, eventually leading to cost-effectiveness. The large-scale production of those nanoparticles or nano-formulations is preferred, which follows the one-pot, green, and environment-friendly products [26, 27].

**Table 1:** The table provides brief information about the different nano-formulations being synthesized using polyphenols, along with their major advantages and synthesis techniques being followed for each formulation

Type of nano-formulations	Material used for making formulations	Advantages of these nano-formulations	Techniques used for the synthesis	Application in wound healing	References
Solid lipid NPs	Bee wax Palm oil Glycerol monostearate Soya lecithin Palmitic oil Soyabean oil	High affinity for hydrophobic compounds Similar constitution as biological membrane Controlled and targeted drug release Increased stability in lipophilic and hydrophilic compounds Non-toxic Easy large-scale production High rate of encapsulation Basic lab equipment required	High-pressure homogenization Solvent emulsification/ evaporation	in vitro*—*HaCaT, human fibroblasts in vivo*—*Female CD-1 mice, HR-1 mice	[78, 81, 82]
Polymeric NPs	Poly D, L-lactic-co-glycolic acid Carbopol Cellulose Collagen Cotton	High chemical and physical stability under physiological conditions Excellent biocompatibility Biodegradability Low toxicity Immunogenicity Easy surface modification Easy synthesis procedures	Nanoencapsulation Chemical modification Ionic gelation Emulsification/solvent evaporation	in vivo*—*Type 2 diabetic mice, Sprague Dawley Rats	[81, 92, 94, 127, 128]
Metallic NPs	Gold Silver Copper Zinc Cobalt Nickel	Adjustable size and shape High specificity Targeted delivery High surface area Sustained drug release	One-pot microwave self-assembly method Laser ablation Pyrolysis Chemical reduction Chemical stabilization	in vitro*—*HaCaT, Hs68, m5S, PHK16-0b in vivo*—*C57BL6/mice, Diabetic Rats	[82, 91, 95, 129, 130, 226] [251]
Non-metallic NPs	Selenium Silicon	Tunable particle size High loading capacity High stability Polar volume Effective release Biocompatible	Co-precipitation Chemical reduction Electrochemical synthesis Stober synthesis Sol–gel method	in vitro*—*HepG2, HaCaT, in vivo*—*Male Kunmimg mice	[83, 84]
Nanogels	Silk Fibroin based hydrogel Silane hydrogel Alginate hydrogel Acrylic acid hydrogel Human defensin-based hydrogel Genistein based nanoemulsions	Biocompatibility Good mucoadhesion Controlled release Enhanced bioavailability High specific surface area	Emulsion polymerization Precipitation polymerization Inverse nanoprecipitation Self-assembly Template assisted polymerization	in vitro*—*HaCaT, BJ-1, Fibroblasts, and Wistar rats in vivo*—*BALB/c Mice, Male Sprague Dawley rats	[85, 86] [103, 112] [126, 131, 141, 146]
Nanofibers	Ferulic acid-loaded nanofibers Caffeic acid-loaded nanofibers Collagen nanofibers Silk	High mesh porosity High specific surface area Effective drug delivery Potent therapeutic activity Biocompatibility	Electrospinning Solution blow spinning Centrifugal spinning Carbon dioxide laser supersonic drawing	in vitro*—*Human fibroblasts, Bacterial strains, in vivo*—*Diabetic rats	[87, 88], [152] [157, 161]
Nanoemulsions	Aglycone based nanoemulsions Resveratrol based emulsion	High encapsulation efficiency High viscoelasticity Bioactive stability Controlled release	High-pressure valve homogenization High-pressure microfluidic homogenization Ultrasonic homogenization Rotor–stator homogenization Spontaneous emulsification Membrane emulsion Phase inversion temperature/composition	in vitro*—*Human fibroblasts and keratinocytes in vivo*—*Sprague Dawley rats Human Patients- 48 male and female participants with diabetic foot syndrome	[78, 89, 225]

### Polyphenol-derived nano-formulations for normal and diabetic wound healing

A wide range of polyphenolic compositions are used to regulate microenvironments of wounds that can be either diabetic or normal and accelerate tissue healing and are discussed in the coming section, along with their mechanism of action.

#### Quercetin

Quercetin is a pentahydroxyflavone that consists of five hydroxy groups placed at 3-, 3′-, 4′-, 5- and 7- positions. Several nano-formulations are made by using quercetin. Choudhury et al. made quercetin-loaded chitosan tripolyphosphate nanoparticles using the ionic gelation method, and due to the increase in density of blood vessels and myofibroblasts, a decrease in the number of inflammatory cells along with deposition and arrangement of collagen fibers, there is better healing in the granulation tissue of NPs treated group of rats [90]. Madhyastha et al. made quercetin functionalized gold NPs that act as free radical scavengers, reduce the intracellular generation of ROS, and help in healing by increasing the migration rates of fibroblasts and keratinocytes by downstreaming the SMAD-7 inhibitory protein by translocating SMAD’s 3 and 4 [91]. Iqbal et al. synthesized curcumin and quercetin-loaded PLGA nanoparticles using different ratios of curcumin and quercetin, which enhanced wound contraction and wound healing time in vivo [92]. It recovers open excision wounds in rats by promoting IL-10, CD31, PCNA, VEGF, and TGF- β1; enhances the proliferation of fibroblasts, increases the microvessel density, collagen deposition, and improves re-epithelialization [93]. Nalini et al. synthesized the carbopol-incorporated quercetin-loaded alginate/chitosan nanoparticles and tested them on open excision wounds in male Wistar rats by applying topically. It decreases the healing period, promotes re-epithelialization and collagen deposition, and enhances antioxidant and antibacterial activity [94]. Badhwar et al. synthesized quercetin-loaded silver nanoparticles, which are embedded in hydrogel matrices and found to be ~ 44 nm in diameter with a spherical shape. The hydrogel is found to have a better antimicrobial activity, with enhanced re-epithelialization rate, eventually resulting in reduced wound gap [95].

#### Catechin and epicatechin gallate

Catechin consists of two benzene rings with a dihydropyran heterocycle with a hydroxyl group at the third position and is known for its antioxidant properties, which help regulate reactive oxygen species [96]. The Air-jet spinning technique prepared a PCL/Catechin/ gelatin film. It was found that the diameter of the outer layer was between 0.3 and 0.6 µm, which prevents the entry of harmful external agents such as bacteria. It thus helps treat wounds due to good healing properties, the ability to enhance the production of IL-10, and decrease the production of IL-1 β and TNF-α [96, 97]. A hydrogel was developed involving hyaluronic acid methacrylate, modified with phenylboronic acid, and conjugated with catechin for glucose-responsive antioxidant action to promote wound healing by reducing inflammatory responses and promoting angiogenesis [98]. Epicatechin gallate (ECG) has the highest anti-oxidant ability out of all catechins and also has anti-inflammatory abilities. It exhibits a better quality of scarring in terms of maturity and collagen orientation because collagen fibers are much more compactly packed [99]. ECG was also tested for treating full-thickness incisional wounds in a diabetic rat model and was found to improve scar formation, wound healing, and other biochemical roles, such as an increase in nitric oxide synthase activity and inducible nitric oxide synthase activity [100]. Epigallocatechin gallate-impregnated wound patches loaded with silver nanoparticles possess both antimicrobial and antioxidant activity, which supports the potential wound healing by promoting collagen deposition, enhanced wound contraction, and vascularization by modulating the cytokines and growth factors at the wound site [101]. The patches were developed incorporating gallocatechin and silver nanoparticles for accelerating diabetic wound healing by increasing the proliferation and inhibition of apoptosis by modulating the Wnt/ β-catenin signaling pathway [102]. Similarly, silk fibroin hydrogels were developed incorporating epigallocatechin for their potent role in scavenging ROS and superior wound healing efficacy [103]. Kim et al. synthesized a collagen sponge incorporated with epigallocatechin for enhanced re-epithelialization and angiogenesis, along with granular tissue reorganization and thickness enhancement by myofibroblasts [104]. Gold nanoparticles were administered along with epigallocatechin gallate and α-lipoic acid and were found to heal the wounds by exhibiting antioxidant and anti-inflammatory effects [105].

#### Luteolin

Luteolin is a 3′ hydroxyflavonoid and tetrahydroxyfavone that exhibits vasoprotective and neuroprotective effects. It inhibits the neutrophil extracellular traps (NET) and ROS. When tested on streptozotocin-induced diabetic rats, it decreased the blood glucose concentration and the expression of MMP-9, IL-1 β, TNF-α, and IL-6 [106]. It increases wound contraction, develops matured collagen fibers and fibroblasts, and improves angiogenesis ability [107]. Different concentrations of luteolin were used to make a luteolin-based ointment for wound healing in diabetic and non-diabetic rat models. A mixture of glycol stearate, propylene glycol, and paraffin was used in 3:6:1 to make 1% and 0.5% w/w concentrations and to study features like re-epithelialization, angiogenesis granular tissue thickness, etc. It was found that ointment shows a significant effect after 14 days in diabetic and non-diabetic rat models [108]. Luteolin ointment was used for wound healing in diabetic and non-diabetic rats [109]. Another ointment was made by mixing luteolin with medical Vaseline and employed for healing cutaneous scald injury by targeting endothelial nitric oxide synthase 3 [110].

#### Curcumin

Curcumin is another secondary metabolite obtained mainly from turmeric and was used to form nano-formulations such as polymeric bandages, collagen films, nanocomposite hydrogels, nanovesicles, chitosan alginate sponge, etc., which acts as a scavenger of free radicals and exhibits anti-oxidant and anti-inflammatory properties. It is a diferuloylmethane having two ferulic acids linked by seven carbon methylene bridges. It also decreases the expression of NF -κ β, TNF-α, IL-1, IL-6, IL-8 [111]. The effect of curcumin was studied on radiation-impaired healing in mice having excisional wounds [215]. Silane hydrogel encapsulated curcumin nanoparticles were tested on burn wounds and exhibited better tissue granulation ability, collagen deposition, new vessel formation, and re-epithelialization [112]. Nanohybrid scaffolds were also made of chitosan incorporated curcumin for better healing activity by enhanced tissue regeneration. These scaffolds had better biocompatibility and sustainable drug availability. It often improves the wound contraction rate by complete epithelialization and forming a thick granular tissue layer [113]. Liu et al. prepared MMP9-responsive and thermos-sensitive hydrogel encapsulated curcumin nanoparticles for diabetic wound healing as particles exhibit anti-oxidant activity and accelerate cell migration [114]. Similarly, Kamar et al. made hydrogel-loaded curcumin nanoparticles for treating excisional skin wounds in rats with type 1 diabetes. It enhances wound healing by doing complete re-epithelialization, increasing collagen deposition for dermal reorganization, and forming intact dermo-epidermal junction [115].

#### Kaempferol

Kaempferol is a flavonol with a backbone of flavone with a hydroxyl group at three positions and is used for diabetic and non-diabetic wounds. The ointments were made using glycol stearate, propylene glycol, and liquid paraffin. The best results were obtained after 14 days in diabetic excisional and non-diabetic incisional wounds treated with 1% w/w ointment [116]. It also assists in bone healing in the standardized mouse tibia fracture model. It accelerates bone formation by initiating bone remodeling and callus formation after 21 days of fracture [117]. It is also used for protecting burn-induced skin injuries by decreasing the expression of TNF-α [118]. A blend was made using chitosan and polyhydroxybutyrate, on which the kaempferol nanocrystals were loaded to check their potential antibacterial activity against twelve different bacterial strains to ensure infection-free wound recovery [119].

#### Resveratrol

Resveratrol is a natural non-flavonoid polyphenolic compound having two polyphenols. Carboxymethyl cellulose-based wafers enriched with resveratrol help in accelerating wound healing [120]. Resveratrol makes the regenerated skin layers more complete, lowers the inflammatory response, and up-regulates nuclear Nrf2 on cutaneous burn injury in diabetic rats [121]. It increases VEGF expression and decreases PDGF expression [122]. It is a well-known antioxidant that assists in scarless healing and acts as an anti-aging agent by preventing skin photoaging [123]. Hyaluronic acid-functionalized nanoparticles delivered curcumin and resveratrol in managing diabetic wounds [124]. Microparticles were synthesized using hyaluronic acid and dipalmitoylphosphatidylcholine and loaded with resveratrol for testing on normal skin fibroblasts. It was found that they increased cellular proliferation and decreased cell oxidation by GSH/GSSHG (Total glutathione/oxidized glutathione) [125]. Comotto et al. synthesized alginate hydrogel dressings loaded with natural antioxidants curcumin and resveratrol and found that none of them were toxic to the keratinocyte cells and increased the cell viability essential for promoting healing [126]. Meng et al. synthesized different nano-formulations involving the resveratrol conjugated with collagen and bacterial cellulose and tested them on human adipose stem cells and determined that the formulations promote cell growth and stem cell attachment by creating a biocompatible environment [127]. Abbas et al. developed resveratrol and naringenin conjugated with β-sitosterol formulation and performed the scratch assay on human fibroblasts, indicating a non-toxic nature and best wound closure rates [132]. Gokce et al. developed resveratrol-loaded hyaluronic acid and dipalmitoylphosphatidylcholine microparticles in the dermal matrix and were found to promote re-epithelialization, collagen fibers accumulation, wound healing in diabetic conditions, and decreased SOD and GPx [133]. A polymer sponge with chitosan-sodium hyaluronate and resveratrol was synthesized by Berce et al. [134].

#### Anthocyanin

Anthocyanin is poly ethoxy or polyhydroxy derivative of 2- phenylbenzophyryllium. Anthocyanins from black soybean seed coats enhance wound healing in Sprague- Dawley rats. It inhibits the translocation of NF -κ β, increases VEGF and CD31, and decreases TSP1 [135]. Anthocyanin complex niosome gel was used to treat oral wounds. It enhances the viability of scratched cells, cell migration, and nuclear elongation. It also increases the expression of collagen, fibronectins, and laminins. Wound healing is accelerated in the presence of anthocyanins as they decrease wound size and pain and improve the quality of life [136]. Blueberry anthocyanin-loaded hydrogel in the injection form was synthesized to promote wound healing in the full-thickness wound by accelerating epithelial and tissue regeneration, promoting collagen deposition and angiogenesis [137]. Silk sericin-encapsulated anthocyanin hydrogel exhibited enhanced bioactivity, ROS scavenging, and wound healing by modulating cytokines and growth factors [138]. Carboxymethyl cellulose and hyaluronic acid-modified hydrogel loaded with blueberry anthocyanins were developed and found to promote epithelial and tissue regeneration, collagen deposition, angiogenesis, and anti-inflammatory cytokines secretion [137]. Similarly, another aerogel-like dressing based on carboxymethyl cellulose/ polyvinyl alcohol loaded with red cabbage anthocyanins was developed to prevent wound infection and promote healing [139]. Anthocyanin and honey-incorporated alginate hydrogel were developed by Lotfinia et al. for their role as antibacterial and wound healing dressings that are sensitive to changes in pH and found to promote cell proliferation [140].

#### Vicenin-2

Vicenin-2 belongs to the flavonoid 8-c-glycosides class of organic compounds. It contains carbohydrate moiety linked to the 8-position of a 2-phenylchromen-4-one flavonoid backbone. It helps enhance cell proliferation and migration when treating human dermal fibroblasts. It increases the expression of TGF-1*β* and VEGF and regulates cytokines such as IL-6, IL-1*β*, and TNF-*α.* A hydrocolloid film was made using vicenin-2 incorporated into sodium alginate, which has desirable wound-healing abilities and much better mechanical strength and is used as a wound dressing to promote wound restoration [141]. Another hydrocolloid film made using vicenin-2 helps enhance diabetic wound healing in a dose-dependent way when tested on Sprague Dawley rats. It reduces pro-inflammatory cytokines, mediators, and nitric oxide [141].

#### Genistein

Genistein is a polycyclic compound with two isoflavone skeletons at the C4 carbon atom with a ketone group. Genistein aglycone was used in the incisional model for wound repair and healing in women after menopause; it enhances the tensile strength of the injury [142]. Further, it suppresses superoxides, inducible nitric oxide synthase, and forkhead box O transcription factor 1, thus accelerating refractory wound healing in patients with type 1 diabetes [143]. It enhances wound contraction during the early stages of recovery by reducing oxidative stress through increasing anti-oxidant ability and modulating the expression of pro-inflammatory cytokines. It also lowered hepatic lipid peroxidation [144]. Dipotassium glycyrrhizinate encapsulated genistein enhances corneal and nerve wound healing in diabetic mice by inhibiting HMGB1 signaling due to the downregulation of IL-1 β and IL-6 cytokines [145]. Acrylic acid hydrogel loaded with genistein-based lipid nano-emulsions was developed to promote wound healing by increasing re-epithelialization and angiogenesis and decreasing inflammation and lipid oxidation [146]. Another hydrogel was developed by Mahajan et al. involving keratin from chicken feathers and genistein, which promotes healing by promoting anti-inflammatory cytokines secretion and faster wound closure [147].

#### Apigenin

Apigenin is a flavone substituted at positions 4′-, 5- and 7- by hydroxy groups, termed trihydroxyflavone. The apigenin-loaded hydrogel was prepared using gellan gum chitosan. The hydrogel exhibits certain features such as biocompatibility, biodegradability, anti-oxidant activity, and moist nature, which aids in healing in both normal and diabetic wounds. Further, it promotes the remodeling of the extracellular matrix and accelerates wound closure by increasing collagen content [148]. The effect of potassium apigenin was observed on wounds in mice. The treatment with apigenin-based gel improves re-epithelialization, inflammation, and neovascularisation [149]. It suppresses CD40, TNF-α, and IL-6 production by inhibiting the IFN-γ-induced phosphorylation of signal transducers and transcription 1 (STAT1) activators in murine microglia [150]. When given alone, Apigenins were found to promote mesenchymal cell differentiation and accelerate bone fracture healing by modulating the Wnt/ β-catenin signaling pathway when tested on Sprague Dawley rats [151].

**Fig. 3:**
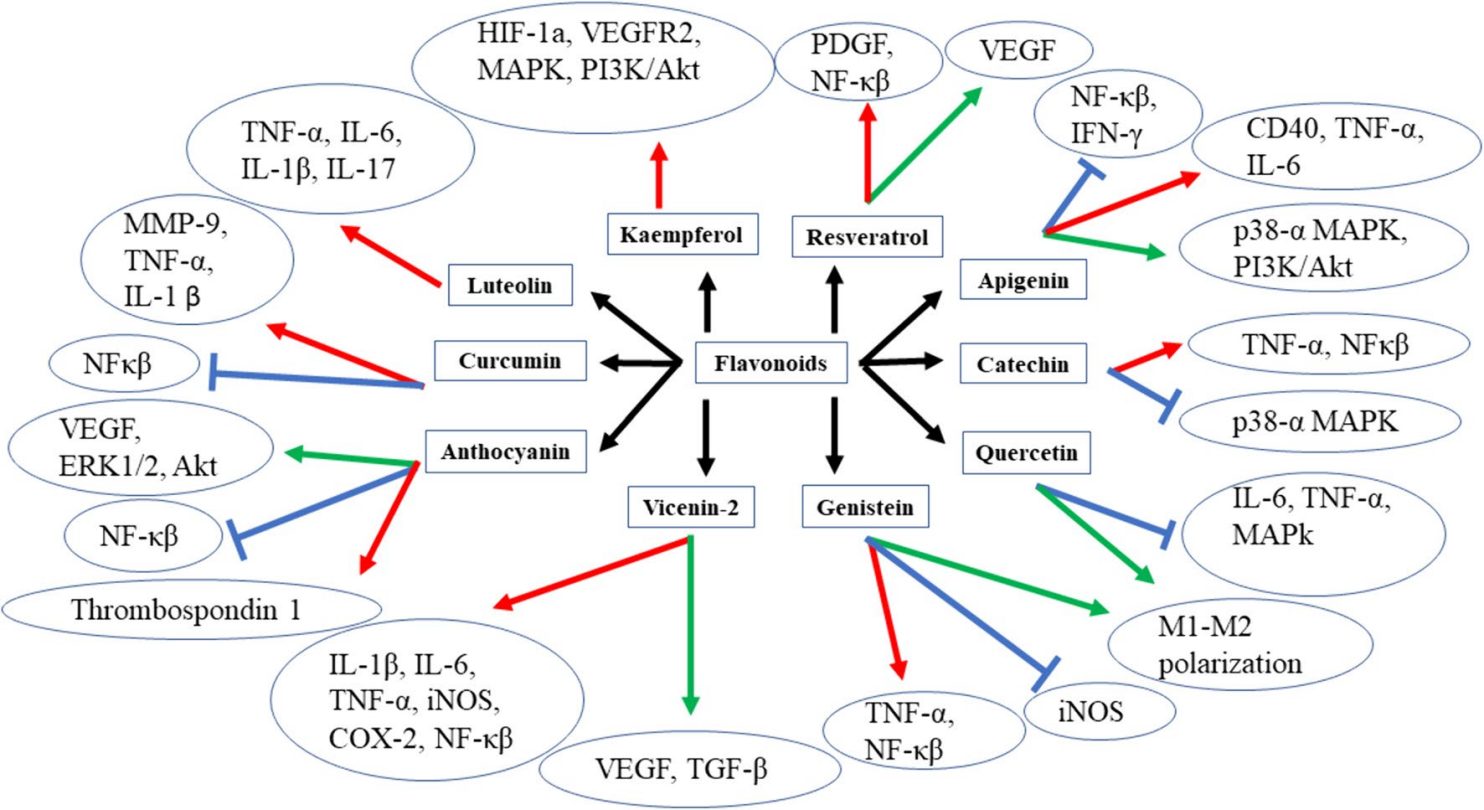
The figure explains the mechanism of different flavonoid-mediated wound healing activities in normal or diabetic wounds. The signs denotes  the decrease  or increase  or inhibition, respectively, of different pro-inflammatory or anti-inflammatory factors due to the effect of flavonoids

The mechanism followed by different flavonoids in treating normal and diabetic wounds is explained diagrammatically in Fig. 3.

#### Caffeic acid

Caffeic acid has phenolic and acrylic functional groups and is classified as a hydroxycinnamic acid. It enhances collagen-like polymer synthesis, inhibits silica-induced ROS generation, and inhibits the release of histamine, which is induced by melittin and arachidonic acid, which prevents vasodilation and thus promotes healing [152]. Caffeic acid phenethyl ester was tested on rats and showed a rapid increase in full-thickness wound healing due to its anti-oxidant and ROS scavenging abilities as it affects NF-κB, NOS2, and NRF2 expression [153]. It increases the nitric oxide and glutathione levels while decreasing malondialdehyde superoxide dismutase in wound tissues, the loss of goblet cells and ciliated cells, and inflammation [154, 155]. PEG/PLGA nanoparticles loaded with caffeic acid phenethyl ester accelerated the wound closure rate, enhanced collagen deposition, proliferation, and angiogenesis, and exhibited antioxidant activity in diabetic rats [156]. PLGA nanofibre-loaded caffeic acid phenethyl ester exhibits antimicrobial and wound healing abilities [157].

#### Ferulic acid

Ferulic acid consists of trans-cinnamic acid with methoxy and hydroxy groups in the phenyl ring at positions 3 and 4. It can be used to cure diabetic wounds because of its specific features such as hypoglycemic effect, free radical scavenging activity, anti-oxidant, anti-bacterial, angiogenic, and neurogenic effects. Ferulic acid-loaded PLGA nanoparticles were synthesized and used for topical and oral treatment of diabetic wounds by increasing epithelialization and hydroxyproline concentration [158]. It is also tested on streptozotocin-induced diabetic rats by excision model. It increases the hexosamine, serum zinc and copper, catalase, glutathione, superoxide dismutase, and nitric oxide levels and inhibits lipid peroxidation, which aids in wound healing [159]. Nanostructure lipid carriers were made to deliver Ferulic acid and *Lavandula* EO, which enhances cell proliferation and migration to treat wounds [160]. Multifunctional nanofibers loaded with ferulic acid were developed by electrospinning for their biocompatibility, antimicrobial efficacy, and accelerated injury healing potential in diabetic rats [161]. Hydrogel was also developed using three distinct materials having antioxidant potential, including polydopamine, ferulic acid, and puerarin, and found to enhance the activity of superoxide dismutase and glutathione peroxidase, decrease the level of reactive oxygen species, and eventually promote wound healing, collagen deposition, and tissue regeneration [162]. Similarly, ferulic acid-loaded chitosan hydrogel was developed to heal corneal wounds by sustained ferulic acid release and anti-inflammatory ability [163]. Another multifunctional bioactive poly(ferulic acid) hydrogel was developed, which is sprayable, injectable, and exhibits self-healing, anti-inflammatory, antioxidant, and angiogenic potential along with ROS scavenging and inhibits the methicillin-resistant staphylococcus aureus [164].

#### Chlorogenic acid

Chlorogenic acid is a cinnamate ester obtained from the condensation of three hydroxy groups of quinic acid with the carboxyl group of trans-caffeic acid. It acts as a potent anti-oxidant due to its role in increasing glutathione, superoxide dismutase, nitric oxide level, and inhibition of lipid peroxidation. Chlorogenic acid or silver sulfadiazine ointment was tested on Wistar rats to treat full-thickness excision wounds and was found to increase collagen synthesis by regulating TGF-1β, TNF-α factors and promoting the proliferation of cells and re-epithelialization [165]. It also enhances hydroxyproline content, angiogenesis, and fibroblast proliferation, decreasing malondialdehyde and nitric oxide levels [166]. Gold nanoparticles were also synthesized using chlorogenic acid, which shows an enhanced inflammatory effect on cell adhesion [167]. The chlorogenic acid and myricetin-3-O-β-rhamnoside isolated from *Parrotia persica* was used for monitoring its wound healing potential [165, 252]. Polyvinyl alcohol hydrogel loaded with chlorogenic acid microspheres significantly promotes epithelialization and collagen fiber production for enhanced wound healing [168]. Another hydrogel involving the self-assembly of chlorogenic acid alone is developed, which exhibits re-epithelialization, collagen deposition, and faster wound closure rates. Further, it suppresses the secretion of proinflammatory cytokines and enhances the section of vascular endothelial growth factor [169].

#### Gallic acid

Gallic acid is a trihydroxy benzoic acid having three hydroxyl groups and one carboxylic acid group attached to the benzene ring. Chitosan nanoparticles loaded with gallic acid were synthesized and linked with collagen fibrin for testing their wound-healing abilities and were found to exhibit enhanced cell migration, fibroblast proliferation, wound contraction, angiogenesis, collagen deposition, and healing [170]. ZnO nanoparticles were incorporated into the gelatin-gallic acid matrix and the conjugate exhibits anti-bacterial activity and act as a multifunctional bioadhesive dressing for healing normal and burn wounds [171]. It activates certain factors that are hallmarks in wound healing, including local adhesion kinases, c-Jun N-terminal kinases, and extracellular regulated kinases [172]. A sodium alginate-based (polyvinyl alcohol-co-acrylic acid) hydrogel loaded with gallic acid has antioxidant, biocompatible,non-irritant, and cutaneous wound healing efficiency [173]. Hexanoyl glycol chitosan conjugated gallic acid was developed and found to promote the regeneration of tissue and wound closure by enhancing the secretion of growth factors and fibroblasts [174]. Another alginate hydrocolloid film dressing loaded with gallic acid exhibited chronic wound healing potential based on its characteristic features [175]. Collagen and hyaluronic acid-based hydrogel mediated with dopamine and gallic acid promotes angiogenesis, cell proliferation, collagen fiber deposition, and inhibits inflammation and ROS [176].

#### Thymol

Thymol is a natural monoterpene, hydride derivative of p-cymene, and carvacrol’s isomer. Lipid nanocarriers encapsulated thymol particles were formed, and the gel was tested on a mouse model, which shows anti-inflammatory and improved healing ability [177]. Combining pluronic F127 and sodium alginate created topical gels into which norfloxacin, ZnO NPs, and thymol were incorporated. Thus, the formulations are used to treat bacteria-infected, bleeding wounds [178] as thymol decreases the expression of TNF-α, IL-6, and IL-1β, attenuating lipoperoxidation and reducing inflammation [179]. Thymol-encapsulated gelatin methacryloyl-based nanoniosomes upregulated the expression of specific growth factors and matrix metalloproteinases and exhibited potential antimicrobial activity against Gram-negative and positive bacteria [180]. Thymol-loaded alginate microparticles were incorporated with chitosan–gelatin films, significantly promoting collagen deposition, epithelialization, skin regeneration, and antibacterial activity [181]. Thymol-enriched bacterial cellulose hydrogel promotes the growth of fibroblast cells and faster wound closure with low toxicity for third-degree burn wound repair [182, 203]. Thymol-loaded Eudragit RS30D cationic nanoparticles-based hydrogels were developed for accelerated wound closure, skin retention, and antibacterial potency [183].

#### Carvacrol

Carvacrol is a 5-isopropyl-2-methyl phenol derived from p-cymene hydrides. Polycaprolactone-incorporated carvacrol particles were formed for the in-site delivery of drugs and for treating the infected wound by enhancing the antimicrobial activity [184]. Carvacrol-incorporated chitosan films were tested on Wistar rats to increase wound healing by inhibiting the expression of IL-4, IL-17, IL-1β, COX-2, and TNF-α [174] and promoting the secretion of IL-10 [185]. Carvacrol-loaded phytosomes decreased the wound area, enhanced cellular proliferation, and induced collagen fiber deposition [186]. Water-soluble carvacrol prodrugs and hyaluronic acid formulations accelerate cell migration and modulate anti-inflammatory cytokines for effective wound healing [187].

#### Tannic acid

Tannic acid is the hydrolyzable tannin having polyphenolic molecules in the center, such as glucose and several hydroxyl groups formed by the esterification of gallic acid or hexahydroxydiphenic acids. Tannic acid-modified silver nanoparticles with better epithelialization, angiogenesis, wound closure, and granulation tissue formation were prepared, enhancing wound healing by increasing the release of VEGF, PDGF β, and TGF-β [188]. Tannic acid cross-linked collagen scaffolds were made for treating wounds, and they increase the protein level of P-Erk 1/2 and stimulate the basic fibroblast growth factor (bFGF) [189]. Tannic acid and hyaluronic acid-modified thin films were developed to promote the wound healing potential antimicrobial and antioxidant activity [190]. Acid-loaded chitosan alginate scaffold accelerates wound closure and tissue regeneration by promoting fibroblast migration [191]. Chitosan/gelatin@tannic acid cryogels loaded with silver nanoparticles were developed by Xu et al. for accelerated wound healing and antibacterial activity [192].

**Fig. 4:**
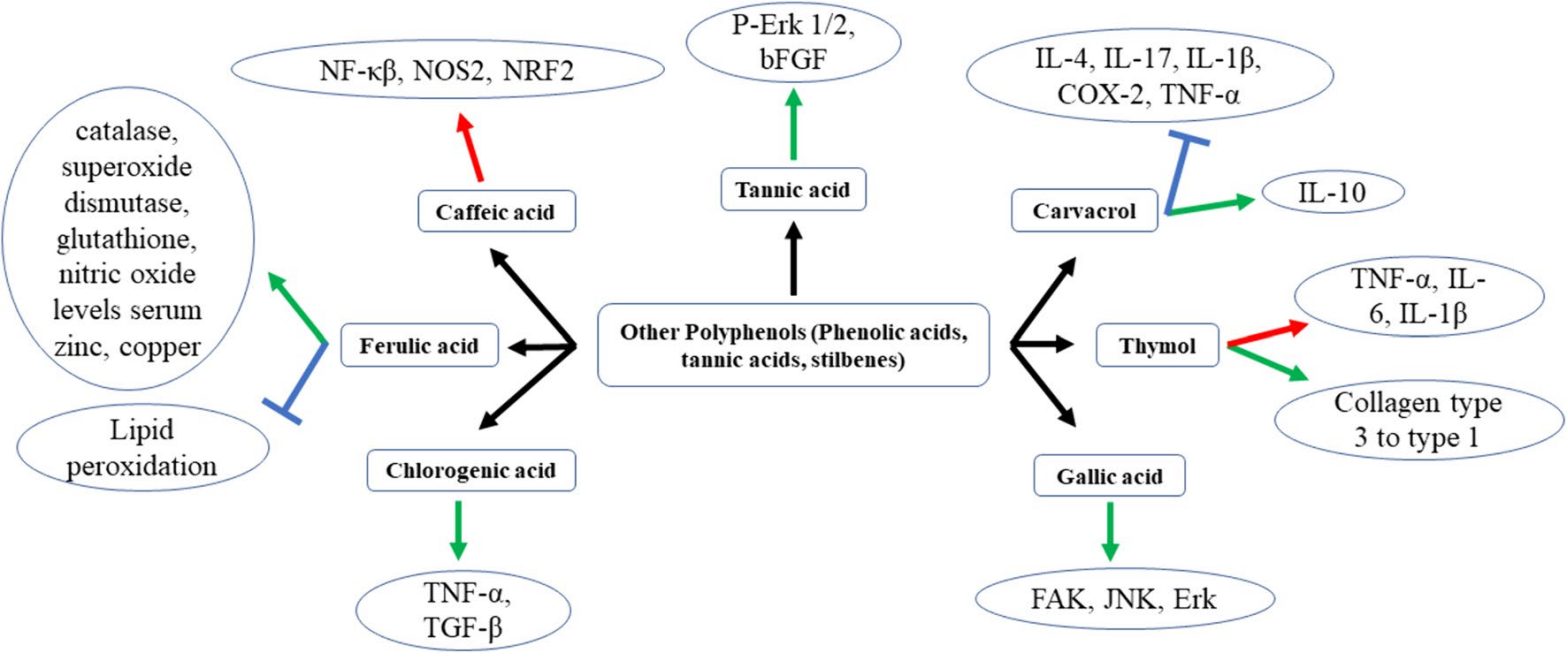
The figure depicts information about the mechanism of different phenolic acids, tannic acids, and stilbenes-mediated wound healing activity in normal or diabetic wounds. The signs respectively denotes  a decrease  or increase  or inhibition of different pro-inflammatory or anti-inflammatory factors due to the effect of phenolic acids, tannic acids and stilbenes

The mechanism followed by all the phenolic acids, tannic acids, and stilbenes to cure wounds is described diagrammatically in Fig. 4.

#### Some other polyphenols

Pomegranate peel is a rich source of polyphenols. It is modified into a gel-like substance called pomegranate peel polyphenol gel (PPP gel), which was further used to treat diabetic wounds in rats. It was found that the wound closure rate is very fast, causing 90% closure in 14 days, as the PPP gel enhances fibroblasts, regeneration of collagen, vascularisation, epithelialization, and mRNA expression of TGF-β1, VEGF, and epidermal growth factor (EGF) [193]. Similarly, polyphenols of green tea also have therapeutic properties in treating wounds. Thus, tea polyphenol nanospheres were synthesized and encapsulated by PVA/alginate hydrogel [207]. They help heal diabetic wounds by inhibiting the NF -κ β expression by inhibiting phosphatidylinositol-3 kinase (PI3K)/ protein kinase B (Akt) signaling pathway, and the damage is healed within five days of treatment [194]. When applied topically on the excised wound, quercetin is sufficient to recover it in diabetic rats [195]. Turmeric enhances wound healing ability in diabetic mice by enhancing glucose sensitivity [196]. A poly-herbal formulation was made using *Hippophae rhamnoides* L., *Aloe vera* L., and the ethanol rhizome extract of *Curcuma longa* L., which enhances angiogenesis and up-regulates VEGF, thus increasing wound contraction in both normal and diabetic chronic wounds [197]. All the nano-formulations obtained from polyphenols by the green method and their mode of action, biological activity, and other physical properties are briefly discussed in Table 2.

**Table 2:** The table provides information about the different polyphenols used to form nano-formulations effective for normal and diabetic wound healing, along with their source, properties, biological activity, and mechanism of action

Name	Structure	Source	Physical properties	Biological activity	Limitations/Drawbacks	Formulations	Tested on	Mechanism of action
Quercetin		Apple, Broccoli, Raw onions, Spinach, Black tea leaves, Green tea leaves [198]	Slightly polar but highly soluble in oil [199, 200]	Antioxidant, Antiviral, Anti-inflammatory, Antibacterial, Anticarcinogenic [201]	Low water solubility due to hydrophobic groups, poor skin penetration, and short half-life (8.8 min)	Quercetin loaded chitosan tripolyphosphate NPs (360 nm) [202]	Wistar rats [202]	Inhibits inflammatory cytokines such as IL-6, TNF-α, induces HO-1 [203] Modulates macrophage polarization from M1 to M2 phenotype [204] Inhibits MAPK pathway [205] Enhances cell migration and proliferation by TGF-β1 dependent SMAD signaling pathway [91] Enhances the bioavailability of curcumin and quercetin, which further enhances wound healing potential [92]
						Quercetin functionalized gold NPs (47 nm) [91]	m5S (Mouse skin fibroblasts) And PHK16-0b (Human keratinocytes) [91]	
						Quercetin loaded PLGA Nps (< 250 nm) [92]	Rabbit's thoracolumbar area [92]	
Catechin		Cocoa, Grape, Apple, Apricot, Cherry, Tea, Red wine [206]	Hydrophilic	Anticarcinogenic, Antitumorigenic, Antimutagenic, Chemo-preventive, Antiproliferative, Antiinflammatory, Antioxidant, Antidiabetic, Antiallergic, Antihypertensive, Antiplatelet, Anti-obesity, Hypocholesterolemic, Neuroprotective [207]	Low stability at pH above four and under sunlight, low bioavailability	PCL/( +)-catechin/gelatin film (0.3–0.6 µm) [96]	NIH/3T3 cells [96]	Decreases TNF-α secretion reduces NFκB activity [208] Inhibits p38-α MAPK activity [209]
						Hyaluronic acid and phenylboronic acid modified hydrogel linked with catechin [98]	Mice [98]	
						Patches incorporating gallocatechin and silver nanoparticles [102]	Diabetic Rats [102]	
Epicatechin gallate		Green tea [210]	Hydrophilic	Antioxidant, Anti-inflammatory, Free radical scavenging [211]	Low stability in basic pH, and high sunlight, low bioavailability	Epicatechin gallate particles [211]	Male Sprague Dawley rats [211]	Upregulates VEGF protein expression [211] decrease the production of the pro-inflammatory cytokines IL-1β and TNF-α enhance the production of the anti-inflammatory cytokine IL-10 [212]
						Silk fibroin hydrogel loaded epigallocatechin [103]	Male Sprague Dawley rats [103]	
						Collagen sponge incorporated epigallocatechin [104]	Type 2 diabetic mice [104]	
						Gold nanoparticles administered with epigallocatechin and α -lipoic acid [105]	HaCaT, Hs68, and mice [105]	
Luteolin		Broccoli, Pepper, Thyme, Celery [213]		Antioxidant, Antimicrobial, Antiinflammatory [214]	Poor solubility in cold water but slightly soluble in hot water, low biocompatibility, high cost	0.5 and 1% w/w ointment with glycol stearate: propylene glycol: liquid paraffin (3:6:1) [108]	Diabetic and non-diabetic rats [108]	Suppresses TNF-α, IL-6, IL-1β, and IL-17 [215]
						Luteolin ointment [109]	Male Wistar rats [109]	
						Luteolin mixed with medical Vaseline [110]	Sprague Dawley rats [110]	
Curcumin		Turmeric [216]	Hydrophobic, Soluble in acetonitrile, chloroform, ethyl acetate, ethanol, methanol, DMSO, etc. [217]	Antimicrobial, Antiinflammatory,Antioxidant [218, 219]	Low stability and poor gastrointestinal absorption, poor solubility due to hydrophobic nature, gets inactivated during liver metabolism, leading to low bioavailability	Silane hydrogel encapsulated curcumin nanoparticles [112, 115]	Dorsal hairs of BALB/c mice [112]	Scavenger of free radicals TGF-β1 and its receptors toll interleukin receptor (tIrc) and functioning [220] Decreases proinflammatory cytokines expression like (IL)-1beta, matrix metalloproteinase-9 (MMP-9), and TNF-α [221] Inhibit the functioning of NFkβ by reducing the activity of AKT, IKK, and PI3K kinases [222]
						Curcumin-loaded chitosan nanoparticles [113]	Rats [113]	
kaempferol			Soluble in hot alcohol, ether, or alkalies, insoluble in benzene, slightly soluble in chloroform	Anti-cancer, Antiproliferative, Antibacterial, Antiinflammatory, Antioxidant [116]	Low water solubility due to the presence of diphenyl propane structure, which gives it hydrophobic characteristics, chemical instability, and extensive metabolic processing, causes low bioavailability	1% w/w kaempferol ointment [116]	Diabetic and non-diabetic rats [116]	Suppress activation of hypoxia-inducible factor-1a (HIF-1a), vascular endothelial growth factor receptor 2 (VEGFR2) via extracellular signal-regulated kinase (ERK)/p38 MAPK and phosphoinositide 3-kinase (PI3K)/Akt/mechanistic target of rapamycin (mTOR) signaling pathways in endothelial cells Decreased angiogenesis was observed in non-diabetic excisional wounds [116] Type III collagen, the major component of the granulation tissue, is replaced by collagen type I, which is the main structural component of the dermis, during wound healing [223]
						Kaempferol nanocrystals loaded polyhydroxy butyrate/chitosan blend [119]	12 Bacterial strains [119]	
Resveratrol		Grape skins, Peanuts, Mulberries, and Red wine [224]	are Soluble in organic solvents such as DMSO, DMF, and ethanol	Antioxidants, Anti-inflammatory, Cardioprotective, Cytoprotective, Anti-cancer, Hepatoprotective [122]	Low water solubility due to its structure, which further affects its absorption rate and low bioavailability due to extensive metabolism in the liver and intestine	Carboxy-methyl cellulose-based wafers incorporated with resveratrol-loaded cellulose acetate butyrate nanoparticles [120]	Rats [120]	Increased VEGF expression and decreased expression of PDGF [122] Reduces imiquimod-induced toll-like receptors (TLR) TLR7, TLR8, and TLR9-mediated psoriatic inflammation in animal models via interfering with NF-kB activation [123]
						Hyaluronic acid-functionalized NPs [124]		
						Polymer sponge having chitosan-sodium hyaluronate and resveratrol [134]	Male mice [134]	
						Electrospun scaffold loaded with resveratrol [225]	C57BL6/mice [225]	
						Collagen-conjugated and bacterial cellulose-conjugated scaffolds loaded with resveratrol [127]	Sprague Dawley Rats [127]	
						Bimetallic Au@AgNPs modified with resveratrol [226]	C57BL6/mice [226]	
						Resveratrol loaded host guest Gelatin hydrogel [227]	Sprague Dawley rats [227]	
						Resveratrol-loaded peptide hydrogel [228]	Sprague Dawley rats [228]	
						Resveratrol and hyaluronic acid-based dermal matrix [229]	48 male and female participants with diabetic foot syndrome [229]	
						Resveratrol cream [230]	20 healthy human volunteers [230]	
						Hyaluronic acid and dipalmitoylphosphatidylcholine-based microparticles loaded with resveratrol [125]	Normal Skin derived fibroblasts [125]	
						Resveratrol combined with Sitosterol [132]	Human fibroblasts [132]	
						Alginate dressings with resveratrol and curcumin [126]	Human keratinocytes [126]	
Anthocyanin		Blackberry, Blueberry, Chokeberry, Cranberry, Elderberry, Raspberry, and Strawberry [231]	Highly soluble in water [232]	Antioxidant, Antiinflammatory [233]	Reduced stability and sensitivity due to several factors like temperature, light, oxygen, and pH	10% w/w anthocyanin in mucoadhesive gel [234]	Rats [234]	Increase VEGF expression Decrease thrombospondin 1 Inhibits translocation of NF-κB (p65/ p50) and its phosphorylation [233] activates signaling pathways, including the extracellular signal-regulated protein kinases 1 and 2 (ERK1/2) and Akt Inhibited the phosphorylation of I*κ*B*α*
						Niosome gel containing anthocyanin clusters [131, 235]	Rats [235]	
						Silk sericin encapsulated anthocyanin hydrogel [133]	Mice [133]	
						Blueberry anthocyanins loaded within hydrogel [134]	Full-thickness rat model [134]	
						Red cabbage anthocyanin-based aerogel [135]	BJ1, *E. coli*, *S. aureus* [135]	
						Anthocyanin and honey incorporate alginate hydrogel [136]	L929, *E. coli*, *S. aureus* [136]	
Vicenin-2		Medicinal plants such as *Urtica circularis and Artemisia capillaris* [236]	Soluble in chloroform, dichloromethane, ethyl acetate, dimethyl sulphoxide, acetone	Antioxidant, Antiinflammatory, Anti-cancer, Hepatoprotective, Antidiabetic, Antihyperglycemic [237]	Low bioavailability due to low aqueous solubility, intestinal barrier, and negative interactions with other body proteins and molecules in high doses	Vicenin-2 hydrocolloid film (containing sodium alginate) [141]	Sprague Dawley rats	Reduces pro-inflammatory cytokines (IL-1β, IL-6, and TNF-α), mediators (iNOS and COX-2), and nitric oxide (NO) via the NF-κB pathway [141] enhance cell proliferation, migration, and wound contraction via the VEGF and TGF-β mechanism pathways [141]
Genistein		Soy products excluding soy sauce [238]	Low water solubility [239]	Antioxidant, Antiinflammatory [239]	Poor absorption in the body leads to poor bioavailability	Genistein [144]	Mice [144]	decreases levels of inflammatory markers TNF-α and NF-κB in genistein-treated mice [240] shift macrophage phenotype from M1 to M2 [241] inhibit inducible nitric oxide synthase (iNOS) activity and production of cytokines [241]
						Dipotassium glycyrrhizinate encapsulating genistein [145]	Diabetic mice [145]	
						Genistein-loaded acrylic acid hydrogel [146]	HaCaT and Wistar rats [146]	
						Hydrogel based on keratin and genistein [147]	Mice [147]	
Apigenin		Artemisia, [242] Sideritis, Teucrium, Genista, Tanacdtum [243]	Soluble in ethanol, pyridine, sulfuric acid, and dilute alkalies	Antiinflammatory, Antioxidant [243]	Low bioavailability as it gets deactivated by the acidic pH of the gastrointestinal region	Apigenin-loaded gellan gum-chitosan hydrogels [148]	Wistar rats [148]	promotes different anti-inflammatory pathways, including p38/MAPK and PI3K/Akt [244] inactivate (NF-κB) [245] suppresses cluster of differentiation 40 (CD40), tumor necrosis factor (TNF-α), and IL-6 production via inhibition of interferon-gamma (IFN-γ)-induced phosphorylation of signal transducers and activators of transcription 1 (STAT1) in murine microglia [150]
						Potassium apigenin and verbena extract-based gel [149]	SKH-1 Mice [149]	
Caffeic acid		Honey bee propolis [246]	Soluble in hot water and cold alcohol	Anti-inflammatory, Antioxidant, Immunomodulatory [157]	Due to its non-polar nature, it is slightly soluble in water	Caffeic acid phenethyl ester [247]	Rats [247]	stimulated collagen-like polymer production inhibited histamine release stimulated by melittin and arachidonic acid in RBL 2H3 mast cells [157] affect NF-κB, NOS2, and NRF2 expression [153]
						PEG/PLGA nanoparticles loaded caffeic acid phenethyl ester [156]	Diabetic rats [156]	
						PLGA nanofibers loaded caffeic acid phenethyl ester [157]	Human fibroblasts, Bacterial strains [157]	
Ferulic acid		Whole grains, Spinach, Parsley, Grapes, Rhubarb, Wheat, Oats, Rye, Barley [248]	Highly soluble in water	Antioxidant, Anti-inflammatory, Antimicrobial, Anti-cancer, Anti-arrhythmic, Antithrombotic, Antidiabetic, Immunostimulant [249]	Poor water solubility, poor intrinsic dissolution rate, and poor permeability cause low bioavailability	Ferulic acid poly (lactic-co-glycolic acid) nanoparticles [158]	Diabetic rats [158]	inhibited the lipid peroxidation elevated the catalase, superoxide dismutase, glutathione, and nitric oxide levels along with the increase in the serum zinc and copper levels [159] Decreases epithelialization period Increases hydroxyproline ferulic [250]
						Nanofibers loaded with ferulic acid [161]	Diabetic rats [161]	
						Hydrogel using polydopamine, ferulic acid, and puerarin [162]	Human periodontal ligament stem cells Sprague Dawley rats [162]	
						Ferulic acid-loaded chitosan hydrogel [163]	Rabbit [163]	
						Multifunctional bioactive poly(ferulic acid) hydrogel [164]	Raw 264.7 macrophages Methicilin resistant staphylococcus aureus [164]	
Chlorogenic acid		Coffee beans Green tea [166] Apples, Carrots, Coffee Beans, Kiwi, Plums, Potatoes, Tea, Tobacco leaves, and Tomatoes [251]		Antioxidant, Antiinflammatory, Radioprotective, Antiulcerogenic, Analgesic ^2^	Poor water solubility causes sub-optimal bioavailability, chemical instability due to molecular configuration, and unstable moieties	1% chlorogenic acid or silver sulfadiazine ointment [165]	Rats [165]	Increased rates of epithelialization Improved cellular proliferation Increased tumor necrosis factor-α levels Upregulated transforming growth factor-β1 [66, 165]
						Chlorogenic acid microspheres loaded polyvinyl alcohol hydrogel [168]	NIH3T3 cells and Rabbit [168]	
						Chlorogenic acid-based hydrogel [169]	HUVEC cells, HaCaT cells, Rat [169]	
Gallic acid		Blueberries, Apple, Walnuts, Watercress	Sparingly soluble in water, soluble in oxygenated solvents	Antioxidant, Antiinflammatory, Analgesic, Neuroprotective, Anti-cancer, Antidiabetic [253]	Poor absorption and rapid elimination cause low bioavailability	Gallic acid conjugated hexanoyl glycol chitosan [174]	BALB/c mice [174]	Increased level of FAK activates JNK and Erk [172]
						Chitosan- copper-gallic acid nanocomposite [254]	Mouse [254]	
						Sodium alginate-based (polyvinyl alcohol-co-acrylic acid) hydrogel loaded with gallic acid [173]	Bacteria and rats	
						Dopamine and gallic acid mediated Collagen and hyaluronic acid hydrogel [176]	Rat [176]	
						Agarose and gallic acid modified hydrogel [255]	Bacterial strains [255]	
Thymol		Thymus, Monarda, Origanum [256]	Soluble in water, alcohol, chloroform, ether, and sparingly soluble in glycerol	Antiinflammatory, Anticancer, Antibacterial [257]	Poor aqueous solubility and susceptibility to oxidation make it unstable	Collagen-based thymol films [258]	Male and female adult Wistar rats [258]	Decrease the expressions of TNF-α, IL-6, and IL-1β Attenuate the lipoperoxidation and reduce inflammation [179] Promote the complete replacement of type III to type I collagen in 14 days
						Bacterial cellulose hydrogel containing thymol [177]	Female albino Wistar rats [177]	
						Thymol-encapsulated gelatin methacryloyl-based nanoniosomes [180]	Human dermal fibroblasts [180]	
						Thymol-loaded alginate microparticles incorporated with chitosan–gelatin films [181]	L929, Rats [181]	
						Thymol-loaded Eudragit RS30D cationic nanoparticles-based hydrogel [183]	BJ-1, Fibroblasts, BALB/c Mice [183]	
Carvacrol		Oregano, Thyme, Spanish origanum, Black cumin, Summer and Winter savory [259]	Slightly soluble in water, soluble in ethanol, ether, and alkalis, very soluble in acetone	Antibacterial, Antifungal, Antiinflammatory, Analgesics [174]	Poor water solubility due to hydrophobic groups attached	Carvacrol incorporated chitosan films [260]	Wistar rats [260]	inhibit the expression of IL-4, IL-17, and IL-1β, COX-2, and TNF-α [174, 185] promoted the increase of IL-10 [185]
						Carvacrol-loaded pyrosomes [186]	Male albino rats [186]	
						Water-soluble carvacrol prodrugs and hyaluronic acid formulations [187]	HaCaT [187]	
Tannic acid		Galls of Rhus and Quercus	Very soluble in alcohol, acetone, insoluble ether, benzene, chloroform, and carbon disulfide	Antioxidant, Antimicrobial, Antiviral, Antiinflammatory [261]	poor lipid solubility causes poor penetration and leads to low bioavailability	Tannic acid-modified silver nanoparticles [188]	Mouse [188]	the protein level of P-Erk 1/2 was increased stimulate the basic fibroblast growth factor (bFGF) [189]
						Tannic acid cross-linked collagen scaffold [262]	Rats [262]	
						Hyaluronic acid and tannic acid-modified films [190]	Human epidermal keratinocytes and bacteria [190]	
						Tannic acid-loaded chitosan alginate scaffolds [191]	Sprague Dawley rats [191]	
						Chitosan/gealtin@tannic acid loaded silver nanoparticles cryogel [192]	Sprague Dawley rats and bacterial strains [192]	
						Chitosan and tannic acid-loaded cellulose films [263]	HaCaT [263]	
						Chitosan/ hyaluronic acid and tannic acid modified hydrogel [264]	Rats [264]	
						Tannic acid functionalized 3D nanofibre sponge [265]	L929, BALB/c Mice [265]	
						Chitin/ tannic acid and polyethylene glycol diglycidyl ether-based hydrogel [266]	Sprague Dawley rats [266]	

## Discussion

Skin wound healing consists of definite, orderly phases which are further severely affected by diabetes. It is a complex process that is not fully addressed by the available therapeutic methods for reasons such as slow healing ability, high cost, less target specificity, etc. Moreover, it is challenging to mimic human diabetic wounds, specifically chronic ones, in the available animal models. The preclinical studies are mainly oriented towards monotherapy, even though diabetic wound treatment is a multi-factorial process. Further, most scientists are working on treating initial wound healing stages and not emphasizing the latter stages, thus making it difficult to cure the wound properly. Also, various antibiotics are available, which need to be modified further based on the current needs using modern approaches. Developed nations such as the USA and some European countries have various medical facilities available for treating chronic wounds, but developing and underdeveloped nations like India and Africa are facing critical issues. However, these countries have rich sources of natural products due to enormous biodiversity, which can be explored to synthesize natural, biodegradable, ecologically sound products that can treat wounds by following a multi-factorial approach.

One such natural product is polyphenols, which are secondary metabolites derived from plants and accelerate wound healing by transporting the inflammatory cells to the position of inflammation and curing it. Also, they increase the movement of endothelial cells and fibroblasts, promote proliferation, and exhibit antimicrobial and anti-oxidant features that make them eligible candidates for healing wounds. Further, they have structures similar to hormones, can bind to some specific proteins, can act as a crosslinker, increase mechanical properties, and reduce oxidative stress. Based on these features, research is going on globally to make stable and bioactive micro and nano-formulations using polyphenols, which can be further used to provide controlled and economically sound release at the wound site. From the current review, we have found several polyphenols that exhibit wound healing ability in diabetic and non-diabetic wounds and are efficient in controlling wound infection by preventing the growth of micro-organisms due to their anti-oxidant, anti-bacterial, anti-viral, and anti-inflammatory properties. The polyphenols exhibit antioxidant activity by scavenging the reactive oxygen and nitrogen species, chelating the metal ions, decreasing the mitochondrial respiration, suppressing the lipid peroxidation, and inhibiting the cyclooxygenase and lipoxygenase. Polyphenols exhibit anti-inflammatory activity by regulating different pathways, including the MAPK pathway, NF -κ β pathway, and arachidonic acid pathway. Further, inhibiting the different cascades and pathways, such as phosphorylation of IKK, translocation of P50 and P65, JNK pathway, ERK pathway, and P38 cascade, also promotes the anti-inflammatory potential of these secondary metabolites. Despite the positive aspects of polyphenols in wound healing, they have limitations such as low water solubility, poor stability, and bioavailability, making their utilization difficult for therapeutic activity.

Several nano-formulations are being developed to efficiently deliver and transport polyphenols across the wound site by overcoming these limitations and controlling their release under specific physiological conditions. Solid-lipid nanoparticles are developed as they have a high affinity for hydrophobic compounds and have a similar composition to biological membranes, making phenol transport easy [82]. Polymeric nanoparticles are synthesized due to their higher chemical and physical stability under physiological conditions, which leads to their easy synthesis and surface modification to incorporate polyphenols [120]. Similarly, metallic and non-metallic nanoparticles have tunable size, shape, high surface area, and thus loading capacity for polyphenols [91]. Nanogels, nano-emulsions, and nanofibers have higher polyphenol encapsulation efficiency and exhibit good biocompatibility and degradability, making them potent polyphenolic carriers [98, 116]. All these recent approaches being followed for delivering polyphenols enhance the therapeutic efficacy by improving the stability, water solubility, bioavailability, half-life, biodegradation, and absorption properties of the polyphenols. In the near future, these polyphenol-oriented nano-formulations need to be explored more by focusing on their physicochemical properties, making efficient and cost-effective nano-drug delivery systems using smart polymers, and stabilizing nano-formulations for site-specific delivery, which can even replace the excessive use of antibiotics and enhance healing at different wound stages by safer and efficient therapeutics using nano-encapsulated polyphenols.

## Data Availability

The review focusses on wound care through nanotechnology-mediated green healing nano-formulations. The study is based on through survey of peer-reviewed articles using databases from Web of Science, Scopus, and PubMed by using following key words: Wound healing, diabetic foot ulcers, phases of healing, polyphenols, Classification of polyphenols, role of polyphenols in skin care and wound healing, polyphenol-based nano-formulations, synthesis techniques of green nano-formulations, green nanotechnology, in vitro*, *in vivo, clinical and preclinical studies. No language barrier was applied to the search.
